# Phase I Study of High-Dose l-Methylfolate in Combination with Temozolomide and Bevacizumab in Recurrent *IDH* Wild-Type High-Grade Glioma

**DOI:** 10.1158/2767-9764.CRC-21-0088

**Published:** 2022-01-05

**Authors:** Lucas A. Salas, Thomas G. Stewart, Bret C. Mobley, Chengwei Peng, Jing Liu, Sudan N. Loganathan, Jialiang Wang, Yanjun Ma, Mitchel S. Berger, Devin Absher, Yang Hu, Paul L. Moots, Brock C. Christensen, Stephen W. Clark

**Affiliations:** 1 Department of Epidemiology, Geisel School of Medicine at Dartmouth, Hanover, New Hampshire.; 2 Department of Biostatistics, Vanderbilt University Medical Center, Nashville, Tennessee.; 3 Department of Pathology, Vanderbilt University Medical Center, Nashville, Tennessee.; 4 Department of Medicine, Yale Medical School, New Haven, Connecticut.; 5 Department of Neurosurgery, Vanderbilt University Medical Center, Nashville, Tennessee.; 6 Tennessee Oncology PLLC, Nashville, Tennessee.; 7 Department of Neurosurgery, UCSF, San Francisco, California.; 8 HudsonAlpha, Huntsville, Alabama.; 9 CD Genomics, Shirley, New York.; 10 Department of Neurology, Vanderbilt University Medical Center.; 11 Division of Neuro-Oncology, Vanderbilt-Ingram Cancer Center, Nashville, Tennessee.; 12 Department of Molecular and Systems Biology, Geisel School of Medicine at Dartmouth, Hanover, New Hampshire.; 13 Epiphany Biosciences, San Francisco, California.; 14 Sir Galahad Labs, Nashville, Tennessee.

## Abstract

Isocitrate dehydrogenase (*IDH*) mutations in low-grade gliomas (LGG) result in improved survival and DNA hypermethylation compared with *IDH* wild-type LGGs. *IDH*-mutant LGGs become hypomethylated during progression. It is uncertain whether methylation changes occur during *IDH* wild-type GBM progression and whether the methylome can be reprogrammed. This phase I study evaluated the safety, tolerability, efficacy, and methylome changes after l-methylfolate (LMF) treatment, in combination with temozolomide and bevacizumab in patients with recurrent high-grade glioma. Fourteen patients total, 13 with GBM, one with anaplastic astrocytoma, all *IDH* wild-type were enrolled in the study. All patients received LMF at either 15, 30, 60, or 90 mg daily plus temozolomide (75 mg/m^2^ 5 days per month) and bevacizumab (10 mg/kg every two weeks).No MTD was identified. LMF-treated patients had median overall survival of 9.5 months [95% confidence interval (CI), 9.1–35.4] comparable with bevacizumab historical control 8.6 months (95% CI, 6.8–10.8). Six patients treated with LMF survived more than 650 days. Across all treatment doses, the most adverse events were diarrhea (7%, 1 patient, grade 2), reflux (7%, 1 patient, grade 2), and dysgeusia (7%, 1 patient, grade 2). In the six brains donated at death, there was a 25% increase in DNA methylated CpGs compared with the paired initial tumor. LMF in combination with temozolomide and bevacizumab was well tolerated in patients with recurrent *IDH* wild-type high-grade glioma. This small study did not establish a superior efficacy with addition of LMF compared with standard bevacizumab therapy; however, this study did show methylome reprogramming in high-grade glioma.

**Significance:**

Glioblastoma (GBM) is a primary brain tumor with a poor prognosis. Therapies to date have failed to improve survival. LGGs, with IDH mutations, have increased global DNA methylation and increased survival compared with GBMs. GBMs lack this mutation and have less DNA methylation. Here we show that the DNA methylome can be modified in GBM with LMF. Such treatment might be useful in methylome priming prior to immunotherapy.

## Introduction

In solid tumors, half of the genome contains large blocks of hypomethylated DNA (1). These hypomethylated blocks are found early in tumorigenesis and correspond to large organized chromatin lysine modifications and lamin-associated domains (2–5). Within these large hypomethylated blocks, at promoter regions of specific genes, there is increased methylation of sites called CpG island (CGI) regions. Gliomas with hypermethylated CGIs are associated with improved prognosis and are classified as having a CpG island methylator phenotype (CIMP; refs. 6, 7). In primary brain tumors, glioma CIMP (G-CIMP) is highly associated with mutations in genes encoding isocitrate dehydrogenase (*IDH1/2*), and the *IDH* mutation, when overexpressed in normal astrocytes, can recapitulate the G-CIMP hypermethylation pattern (6–8). G-CIMP tumors have improved prognosis, irrespective of tumor grade, but *IDH* mutations and the G-CIMP epitype are rare in the most common and aggressive glioma, glioblastoma (GBM; ref. 6). In addition to the large blocks of DNA hypomethylation found in the initial tumor, there is additional loss of DNA methylation during tumor recurrence or tumor progression in many cancer types (1, 2). Similarly, hypomethylation occurs during the progression of *IDH*-mutant low-grade glioma (*IDH*-mutant LGG) to secondary (*IDH*-mutant) GBM (9), and during *IDH-*mutant LGG (grade II/III) recurrence, where G-CIMP–high tumors progress to G-CIMP–low tumors with hypomethylation in the CIMP genes (9, 10). Moreover, DNA hypomethylation occurs during *IDH*-mutant secondary GBM recurrence (9, 11). Together, this implies that recurrent or progressing gliomas have loss of DNA methylation (hypomethylation) compared with the primary tumor.

Recent data by Zhou and colleagues have shown that DNA hypomethylation in cancer is mainly found in lamina-associated domains that are located within late-replicating regions of the genome (12). Moreover, it was found that DNA hypomethylation increased with age and tracked the accumulation of cell divisions, and was associated with somatic mutation density. Thus, they proposed a remethylation window model, where replication late in the S-phase provides less time for remethylation of newly synthesized daughter strands during DNA replication, contributing to hypomethylation during tumor growth (12).

It has been shown that treatment with the methyl donor, folate, increased global DNA methylation in cultured glioma cells. Moreover, in a xenograft-induced glioma mouse model, folate increased global DNA methylation and reduced tumor size (13, 14). It is unknown whether DNA hypomethylation occurs with primary GBM progression (*IDH* wild-type) and it is unknown if the epigenome of gliomas can be reprogrammed.

These data provide a rationale for exploring the use of a methyl donor in recurrent high-grade glioma. In this study, we evaluated the safety, tolerability, preliminary efficacy, and DNA methylome dynamics after treatment with the methyl donor, l-methylfolate, combined with temozolomide and bevacizumab in recurrent *IDH* wild-type high-grade gliomas.

## Materials and Methods

### Protocol Objectives

The primary objective of phase I was to determine the MTD of l-methylfolate (LMF) in combination with bevacizumab and temozolomide in patients with *IDH* wild-type recurrent (first recurrence) malignant glioma. The secondary objectives were to assess the objective response and safety profile of LMF in combination with bevacizumab and temozolomide in patients with *IDH* wild-type recurrent MG.

### Patient Eligibility

This protocol (ClinicalTrials.gov # NCT01891747, using the CONSORT guideline) was institutional review board (IRB) approved, and all study patients signed written informed consent. Study patients, 18 years of age or older, must have had histologically proven malignant glioma. Patients must have had genetically confirmed *IDH* wild-type tumor. Patients must have had measurable contrast-enhancing recurrent malignant glioma by MRI within two weeks of starting the treatment. Measurable disease is defined by at least one enhancing lesion accurately measured in at least one dimension greater or equal to 5 mm and patients could have nonmeasurable disease if they had recent surgery for radiographic progression but no patients had recent surgery. They must have recovered from the severe toxicity of prior therapy. Study patients must have had a Karnofsky Performance Status greater or equal to 60. They must have had adequate bone marrow and organ function. Women of childbearing potential were required to have a negative pregnancy test within 10–14 days.

Exclusion criteria include prior treatment within two weeks of entering the study, genetically confirmed *IDH1/2* mutation, HIV positive, or pregnancy. The remaining exclusion criteria were similar to those of previous brain tumor therapeutic clinical trials.

### Trial Design

In this nonrandomized prospective phase I trial with standard 3 + 3 design, study patients received LMF (Alfasigma) orally twice daily in a 28-day cycle and bevacizumab 10 mg/kg every two weeks by intravenous infusion in an outpatient setting on days 1 and 15. In addition, patients received a 5-day regimen of temozolomide at 150 mg/m^2^/day each cycle, and daily 250 mg tablet of vitamin C. Dose escalation involved 3 patients treated at each dose level of LMF (15 mg, 30 mg, 60 mg, or 90 mg) and dose escalation occurred in a stepwise fashion. The 15-mg dose level was a once-a-day dose of 15 mg, whereas subsequent dose levels were twice daily (i.e., 30-mg level, was 15 mg twice daily). MTD was not reached. Patients were continued on therapy until there was disease progression or significant toxicities occurred.

This phase I study was conducted in accordance with the Declaration of Helsinki, International Ethical Guidelines for Biomedical Research Involving Human Subjects (CIOMS), Belmont Report, and the U.S. Common Rule.

### Toxicity Assessment

At each patient visit, both safety and toxicity were evaluated. The Common Terminology Criteria of Adverse Events (CTCAE version 4.02) was used to grade adverse patient events. If patients experienced a grade 4 nonhematologic dose-limiting toxicity (DLT), LMF, and/or bevacizumab and/or temozolomide was stopped (this did not occur). We defined hematologic DLTs as those where platelets were equal or less than 25,000 or if a second occurrence where the platelets fell below 50,0000; the latter would be considered a grade 3. Other conditions that were considered as hematologic DLTs were any event in which there is clinically significant bleeding and a platelet count below 50,000 or an ANC equal or below 1,000/μL or febrile neutropenia.

### Response Assessment

Patients were evaluated every 8 weeks with a contrasted brain MRI. We used the criteria outlined in the Response Assessment in Neuro-Oncology (RANO) for evaluation of changes in tumor (15). Patients were evaluated whether they had a complete response, partial response, stable disease, or progressive disease as outlined by the RANO criteria (15).

### Bevacizumab Cohort

To generate a bevacizumab control group, we performed a retrospective chart review for all patients with recurrent GBM treated at Vanderbilt Medical Center (Nashville, TN). After approval by the Vanderbilt IRB, we identified 50 patients treated between the January 1, 2005 and December 31, 2014. To avoid any selection bias, we had the following inclusion criteria: (i) GBM diagnosis; (ii) treated with only with standard therapy including chemoradiation with temozolomide, followed by adjuvant monthly temozolomide; (iii) first recurrence; (iv) only one surgical resection for initial diagnosis and no further surgery; (v) recurrence had to be greater than 8 weeks from the completion of radiation; (vi) patient had to have signed consent for database participation; (vii) patients had to be treated with bevacizumab therapy at recurrence.

### Survival Statistical Analysis

We defined progression-free survival (PFS) as the time between starting therapy and progression on MRI or death. We defined overall survival (OS) as the time from clinical trial start date to time of death. The Kaplan–Meier method was used to estimate PFS and OS. A median (range) was used to summarize continuous variables and frequencies and percentages were used to describe categorical variables. Any patients that remained alive at the end of the study were censored in the OS calculation.

### O^6^-methylguanine-DNA Methyltransferase Gene Promoter Methylation and *IDH* Mutation Analysis

This protocol is for O^6^-methylguanine-DNA Methyltransferase Gene (*MGMT*) methylation and *IDH* mutation results used on all tumors seen at the Vanderbilt University Medical Center (Nashville, TN) and are not to be confused with the methylation studies involving CpGs described later.

DNA was isolated from formalin-fixed paraffin-embedded (FFPE) patient specimens. Using our AB17900 PCR (Applied Biosystems), methylation-specific PCR analysis was carried out. Methylated MGMT was defined as those tumors with a methylation score greater than 2. *β-actin* was used as a control for copy number. We used multiplex PCR and primer extension to evaluate for *IDH1* and *IDH2* mutations. Products of PCR were fluorescently labeled and analyzed with capillary electrophoresis. The following mutations were tested for *IDH1*: R132G, R132S, R132C, R132H, R132 L, and R132P. For *IDH2*, the following mutations were tested: R140P, R140L, R140Q, R140G, R140W, R172S, R172T, R172M, R172K, R172G, and R172W.

### Surgical and Autopsy Sample Acquisition

All patients provided informed written consent. The use of trial (LMF) patient's initial tumor and autopsy tumor was approved by the Vanderbilt IRB. Controlled deidentified paired tumor samples were obtained after approval from Vanderbilt and UCSF IRBs. In the latter case, deidentified paired GBM tissue was obtained from the UCSF Brain Tumor SPORE Tissue Core (P50CA097257). Where possible initial, recurrent, and autopsy samples were collected during surgical resection (or at autopsy) and were snap-frozen in liquid nitrogen and stored at −80^°^ C. All autopsy samples were fresh-frozen.

### DNA Isolation

Genomic DNA was isolated from fresh-frozen or FFPE samples. DNA was extracted from the samples using the QIAGEN DNeasy kit (Qiagen Inc.) following the manufacturer's protocol at Hudson Alpha. The FFPE samples required the use of a DNA Restoration Kit (Agencourt FormaPure, Beckman Coulter, and Illumina's FFPE Restore Kit, Illumina Inc.) prior to processing.

The isolated genomic DNA was stored at −80°C until use. For every sample, 1 μg of DNA was bisulfite-converted using the Zymo EZ DNA Methylation Kit (ZYMO Research Corporation) according to the manufacturer's instructions. Converted DNA was eluted in a 22 mL elution buffer. DNA methylation level was assessed using the Infinium Human Methylation EPIC Beadchip (Illumina Inc.) at Hudson Alpha according to the manufacturer's instructions. Bisulfite-converted DNA was amplified and then enzymatically fragmented and precipitated. The DNA was then dissolved in hybridization buffer and placed on the Human Methylation EPIC Beadchips. To reduce batch effects, samples were distributed in random blocks and hybridization was allowed to occur for 20 hours at 48°C in Illumina's hybridization oven. Free DNA was then washed away, then the beadchips were processed through a single nucleotide extension following IHC staining (dideoxynucleotides triphosphates; ddNTP) using capillary flow through chambers (Tecan GenePaint automated slide processor). Using the Illumina iScan software and iScan system, a fluorescent signal was captured. Using Illumina GenomeStudio software, background subtraction was completed and intensity data (idat) files were generated, one per channel. A similar process was performed in the The Cancer Genome Atlas (TCGA) datasets, although they only used fresh-frozen samples not requiring the use of the restoration kit; the samples of the TCGA were run using Infinium HumanMethylation450K BeadChips.

### Bioinformatics Analyses

The idat files generated from the Infinium Human Methylation EPIC Beadchip and the Infinium Human Methylation 450 Beadchip (Illumina, Inc.) were imported and preprocessed using the minfi and sesame packages in R. DNA methylation β-values were estimated based on the measured intensities of the two-paired channels (i.e., red and green) and computed as the ratio of the methylated probe intensity, divided by the sum of the unmethylated plus the methylated intensities signals plus an offset of 100 (16, 17). β-values range between 0 and 1 and can be interpreted as a proxy to the proportion of methylated alleles at a specific CpG site. β-values were background corrected using noob and a nonlinear dye correction using sesame. As two different generations of microarrays were combined, we included the microarray slide as a fixed effect to control potential batch effects. Low-quality samples were considered whether the probes showed no statistical difference using an out-of-band sesame array hybridization p-OOBAH detection value (*P*_detection_ > 0.05), or whether the mean bisulfite conversion probes of a specific sample were below 3 SD of the mean bisulfite conversion of all the probes. Probes that were marked as low quality in more than 5% across samples were masked for all the samples, and samples that showed more than 30% of probes with low-quality detection were eliminated from the analyses. Probes marked as CpG loci on the X and Y chromosomes, and those previously documented as polymorphic or cross-reactive were excluded from subsequent analyses (18).

### Genome-wide DNA Methylation Analyses

We implemented a locus-by-locus analysis aimed at identifying differentially methylated CpG sites based on the treatment effect (using the package limma; ref. 19). Briefly, linear mixed effect models were fit to each CpG site separately and modeled β-values of the DNA methylation as the response against the treatment versus the controls. Models were adjusted for chronologic age (years), sex, sample method processing (fresh-frozen or FFPE), microarray slide, and subject as a random effect. Although our examination was exploratory in nature, *P* values were adjusted for multiple comparisons by computing the Benjamini–Hochberg Q values also known as false discovery rate (FDR). As we expected small effect sizes, we ranked the CpG loci according to the FDR, prioritizing those with the biggest magnitude of the DNA methylation differences (absolute Δβ >0.2) for further exploration. All analyses were carried out using the R statistical package, version 4.0.2 (www.r-project.org/).

### DNA Methylation Changes During *IDH* wild-type GBM Recurrence

We identified 15 total matched samples, five from our institutions (Vanderbilt/UCSF, 850K chip), and ten from the TCGA (450K chip) of *IDH* wild-type GBMs with initial tumor and recurrent tumor data. To determine the extent to which these tumors had altered methylomes, we calculated the change in methylation (β value, the methylated fraction at a CpG site) from initial GBM to recurrent GBM at each CpG site in each patient, and identified CpG sites with consistent methylation changes upon recurrence across all patients. We restricted our analysis to shared CpG sites between the two methylation array platforms (450K and 850k) and fitted linear mixed effect models to test the relation of methylation with GBM recurrence.

### Genome-wide DNA Methylation Dysregulation Index

To evaluate global methylation changes between paired samples, we used a modification of the methylation dysregulation index (MDI; ref. 20). Briefly, this modified MDI measure represents the cumulative change from the primary tumor DNA methylation levels in a CpG locus–specific manner calculated by summing the absolute difference in DNA methylation β-values at each CpG between each recurrent tumor sample and the matched primary tumor sample β-value and then divided by the total number of interrogated CpGs. To better reflect scale, MDI was multiplied by 100. The MDI here represents the average change in β-value per CpG in the recurrent tumor sample compared with the primary sample. Therefore, an MDI value close to 0 suggests a similar methylation profile to the matched primary tumor while increasing levels of MDI indicate that the DNA methylome has been deregulated to a greater extent in the recurrent tumor.

### Enrichment Analyses

For the DNA methylation analyses, a test for enrichment was performed using the missMethyl package (21). This algorithm maps the CpG probes to gene IDs and test for Gene Ontology (GO) and KEGG pathways using a hypergeometric test. As in the design of the Illumina arrays, multiple CpGs could be present in the same locus, the test for enrichment is corrected taking into account the bias generated for the number of CpGs per gene in the EPIC or Illumina Human Methylation 450K arrays. The terms obtained in the GO analyses were grouped using REVIGO into hierarchical groups (22).

### Statistical Analysis

All analysis was performed using R version 4.0.2 or higher, using R packages for statistical analysis, DNA methylation, and gene expression preprocessing and analysis including Table 1 (general descriptive statistics), survival (survival analysis), minfi and sesame (DNA methylation preprocessing, quality control, filtering, background correction, and normalization), limma (linear models and linear mixed effect models), q value (FDR adjustments), and missMethyl (hypergeometric test for enrichment). Graphs were generated using R plots, ggplot2, treemap (REVIGO), and heatmap.

**TABLE 1:** Patient characteristics

Characteristics	LMF + bevacizumab + temozolomide	Bevacizumab Cohort	TCGA Cohort	Vanderbilt/UCSF Cohort
n (pathology)	14 (13 GBM, 1 AA)	50 (all GBM)	10 (all GBM)	5 (all GBM)
Age, median (range), years	59 (41–71)	58 (18–75)	60 (36–72)	56 (38–72)
Gender (%)				
Male	86	52	67	25
Female	14	48	33	75
KPS, median (range)	80 (60–90)	80 (50–100)	d	80 (70–90)
Received chemoradiation with temozolomide (%)	100	100	100	100
*MGMT* methylated (%)^a^	33	b	b	b
*IDH* mutated (%)	0	b	0	0
Median exposure to LMF (range), days^c^	192 (46–701)	–	–	–

^a^Nine of the 14 samples had *MGMT* methylation data and all were IDH wild-type.

^b^In the majority of these patients the *MGMT* and *IDH* status were not known.

^c^Days to progression.

^d^KPS is not known for TCGA samples.

Abbreviation: KPS = Karnosfsky performance status.

### Data Availability Statement

All high-throughput data mentioned in the article are publicly available from GEO under accession GSE111627. All data that support the findings of this study are available from the corresponding author upon reasonable request.

## Results

**FIGURE 1:**
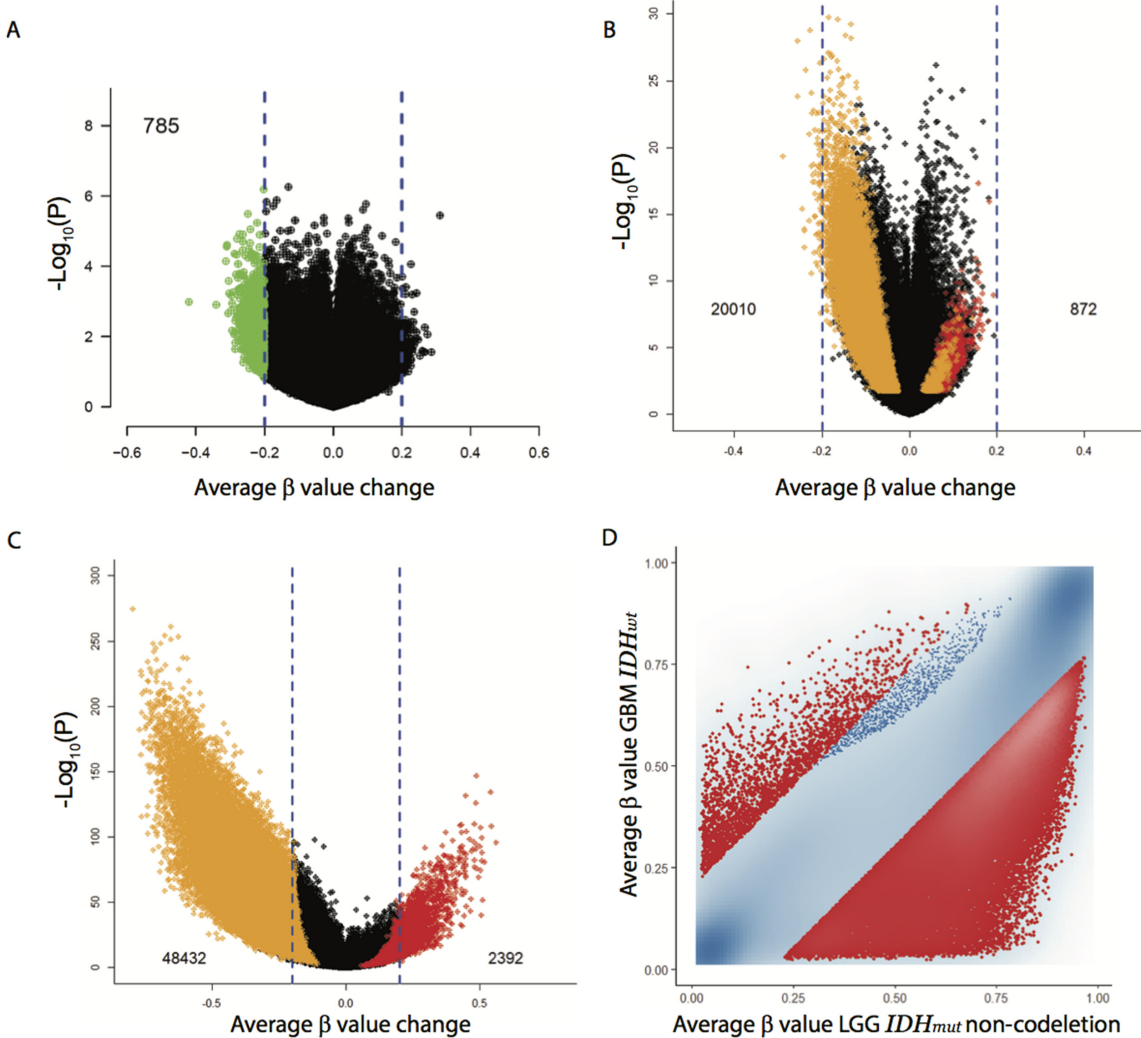
DNA methylation changes during IDH-wild-type (IDH-wt) GBM recurrence. **A,** Volcano plot comparing the average methylation change from primary GBM to recurrent GBM using paired samples from TCGA and from our institutions (450K methylation platform). 785 probes with reduced methylation in recurrent GBM of >0.2 on the average beta scale are shown in green. **B,** Volcano plot comparing overlapping CpGs from GBM to LGG (not specific for IDH) using TCGA methylation data (450K methylation platform). Colored dots represent CpG sites that show significant hypermethylation, FDR<0.05 and Δβ > 0.2, (red dots, total count provided) or hypomethylation, FDR<0.05 and Δβ < −0.2, (orange dots, total count provided) including those that were inconsistent between the model coefficient and the Δβ direction. **C,** Volcano plot comparing overlapping CpGs from GBM (*IDH wt*) to LGG (*IDH mutant*). Colored dots represent as in **B**. **D,** GBM (*IDH wt*) –LGG (*IDH mutant*) scatterplot, each axis representing the average β-value per interrogated CpG, GBM *IDH-wt* on the *y*-axis and LGG *IDH-mutant* on the *x*-axis.

We evaluated the DNA methylome changes of *IDH* wild-type GBM at recurrence and compared baseline methylation status between GBM and LGG and especially *IDH* wild-type GBM and *IDH*-mutant LGG. First, we compared the DNA methylomes between matched samples (initial tumor at diagnosis to the recurrent tumor from the same patient) of primary (*IDH* wild-type) GBMs, using Illumina's Infinium Human Methylation 450K and Methylation EPIC(850K) Beadchips. We identified 15 total matched samples, five from our institutions (Vanderbilt/UCSF, 850K chip), and ten from the TCGA (450K chip). The clinical, histopathologic, and molecular characteristics of these patients are summarized in Table 1 (third column “TCGA Cohort” and fourth column “Vanderbilt/UCSF Cohort”). We identified 785 CpG sites that were specifically hypomethylated in recurrent GBM (|Δβ|>0.2, or >20% decrease in methylation), more than six times the 124 CpGs with an equivalent increase in methylation (Fig. 1A, green dots: hypomethylated CpGs; ref. 9). Due to the small sample size, the methylation β values did not reach statistical significance after correcting for multiple comparisons.

Using TCGA methylation data, we compared GBM to LGG and found significant hypomethylation in GBM compared with LGG (Fig. 1B, orange dots: hypomethylated CpGs from GBM compared with LGG; red dots: hypermethylated CpGs from GBM compared with LGG). This difference is most apparent when comparing *IDH* wild-type GBMs to *IDH*-mutant LGGs (Fig. 1C and D, orange dots: hypomethylated CpGs from GBM *IDH* wild-type compared with LGG *IDH-*mutan*t*; red dots: hypermethylated CpGs from GBM *IDH* wild-type compared with LGG *IDH-*mutant).

We next investigated whether LMF could reprogram the DNA methylome in recurrent high-grade glioma patients by increasing DNA methylation. Patients with recurrent primary (*IDH*-wild type), high-grade glioma were enrolled in a phase I study and treated with LMF (NCT01891747; patient characteristics, Table 1, first column LMF + bevacizumab + temozolomide). Fourteen patients with recurrent high-grade glioma (13 patients with GBM and one patient with anaplastic astrocytoma) received twice-daily LMF along with standard therapy bevacizumab and temozolomide. The median age of the study population was 59 (range, 41–71) years. Most patients were men. Six patients decided to donate their brains at death.

Overall, the most common AEs were diarrhea in 1 (7%) of 14 patients, reflux in 1 (7%) patient, and dysgeusia in 1 (7%) patient (Table 2). There was no grade 3 or higher toxicity. No laboratory toxicities were appreciated and no MTD was reached.

**TABLE 2:** Toxicity results of LMF treatment in phase 1

Patient#	Cycle	Total dose (LMF)mg/d	Toxicity Category	Toxicity	Grade	Clinically significant	Overall relation	Invest Tx
5	4	30	GI	GI reflux	2	No	Invest Tx	LMF
6	2	30	GI	Diarrhea	2	No	Invest Tx	Vit C
7	1	30	CNS	Dysgeusia	2	No	Invest Tx	LMF
7	3	30	CNS	Dysgeusia	1	No	Invest Tx	LMF
9	1	60	GI	Dyspepsia	1	No	Invest Tx	LMF
9	2	60	Derm	Pruritus	1	No	Invest Tx	LMF
9	2	60	GI	Dyspepsia	1	No	Invest Tx	LMF
9	5	60	GI	Bloating	1	No	Invest Tx	LMF

**FIGURE 2:**
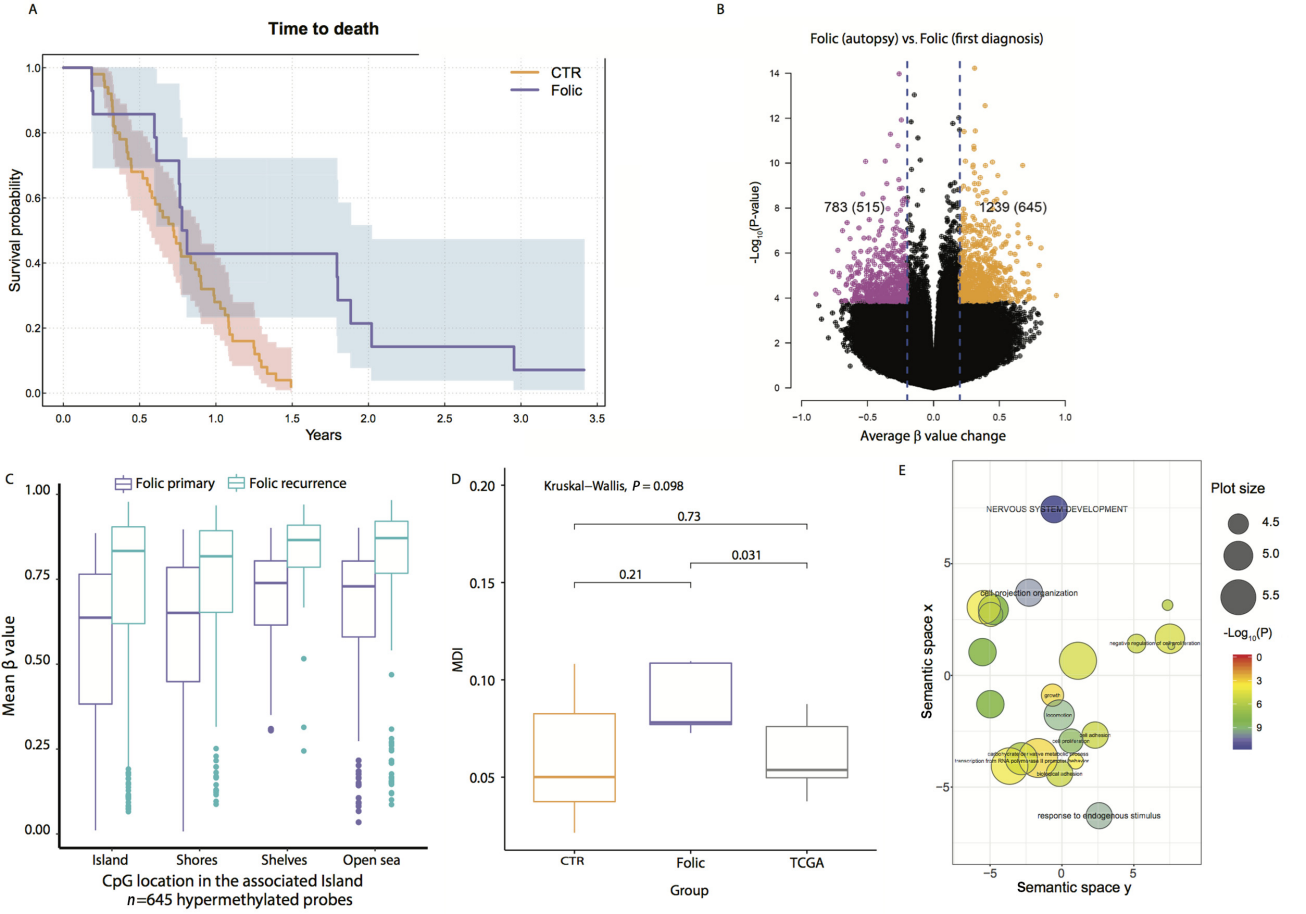
DNA methylation changes in recurrent *IDH*-wt GBM after treatment with LMF. Note: “Folic” is LMF. **A,** Kaplan–Meier curve of OS in LMF-treated patients (blue line) versus historical control group treated with bevacizumab alone (red line). The 95% CI is represented by the blue and red shaded areas. See Supplementary Table S1 for patient characteristics and n value. CTR, bevacizumab treatment; folic is LMF-treated patients. **B,** Volcano plot comparing the average methylation change from initial GBM (at diagnosis) to autopsied GBM (after exposure to LMF at recurrence, measurements were taken from distinct paired samples). Colored dots represent CpG sites that show significant hypermethylation, FDR<0.05 and Δβ > 0.2, (orange dots, total count provided in the parentheses) or hypomethylation, FDR<0.05 and Δβ < −0.2, (purple dots, total count provided in the parentheses) at autopsy. The total CpGs that were FDR<0.05 at any level of Δβ are summarized outside the parentheses. The volcano plot was generated using the 850K-EPIC platform. Folic, LMF treated. **C,** The genomic distribution of the 645 hypermethylated CpGs [red box plot, initial GBM (at diagnosis); blue box plot, autopsied GBM tumor (after exposure to LMF at recurrence)]. Center line, median; box limits, upper and lower quartiles; whiskers, 1.5× interquartile range; points, outliers. Folic, LMF treated. **D,** Comparison of the DNA MDI between LMF-treated patients (Folic), TCGA paired samples (TCGA), and our institutional paired controls (CTR). Box plot elements as in **C**. **E,** Using gene ontology (GO) analysis, we show pathways impacted by the 645 hypermethylated CpGs identified in **B**. GO terms were summarized using REVIGO (http://revigo.irb.hr/). For each class, terms remaining after the redundancy reduction are represented in a two-dimensional space, which summarizes GO terms’ semantic similarities. Semantically similar terms are close together in the plot, but the semantic space units have no intrinsic meaning. Bubble color indicates the *P* value (all terms included had a *P* < 0.05); circle size indicates the frequency of the GO term in the underlying GO database (more general terms have larger plot size). CpG Island: 300 to 2,000 bp in length close to promoters with high GC content; CpG Shores: <2 kb from CpG Island; CpG Shelves: 2–4 kb from CpG Island; Open Sea: >4 kb from CpG Island.

Out of 14 patients treated with LMF, 2 patients had progressive disease (14%), 6 patients with partial response (43%), and 6 with stable disease (43%). LMF-treated patients had a median OS (mOS) of 9.5 months (95% CI, 9.1–35.4) and PFS of 6 months (95% CI, 3.6–10.1) and these were not statistically different from the bevacizumab control cohort (patient characteristics, Table 1, second column, Bevacizumab cohort) with a mOS, of 8.6 (95% CI, 6.8–10.8), and PFS of 4.1 (95% CI, 2.8–6; Fig. 2A; Supplementary Fig. S1). Of the 14 patients treated with LMF, one patient is alive, and 6 patients survived longer than 650 days (656, 658, 689, 739, 1,080 days), whereas two survived less than 73 days (72 and 69 days). Six of the trial participants (all GBM) donated their brains at death and were available for additional studies; time on LMF of these 6 patients: 46, 76, 108, 365, 459, and 620 days. (Supplementary Table S1).

Using the Illumina 850k Methylation platform, we compared DNA methylation between paired samples, initial tumor to the autopsied tumor from the same patient in the 6 patients that donated thier brains after LMF treatment. We observed increased DNA methylation in the autopsied tumors of LMF-treated patients, with 1,239 CpGs demonstrating significantly increased DNA methylation compared with the initial tumor (Fig. 2B, right volcano plot; black and orange dots). Next, we broadly analyzed the genomic location of the DNA hypermethylation (1,239 CpGs) and found these methylated CpGs associated with the DNA shelf, shore, and open sea, but not within the CGIs (Supplementary Fig. S2). When we restricted our analysis to the most hypermethylated CpGs (645 hypermethylated > 0.2Δβ), we found increased methylation across the genome, including CGIs (Fig. 2C). Furthermore, LMF treatment reversed the DNA hypomethylation/hypermethylation ratio in LMF-treated patients (515 hypomethylated to 645 hypermethylated CpGs) compared with that found in LGG transition to secondary (*IDH*-mutant) GBM (4,343 hypomethylated to 311 hypermethylated CpGs) and primary (*IDH*-wild-type) GBM recurrence (785 hypomethylated to 124 hypermethylated CpGs; ref. 9).

To further highlight the methylation dynamics imparted by LMF treatment, we compared control recurrent GBM groups (TCGA and Institutional samples) to the LMF-treated group using an aggregate measure of methylation alteration, MDI (20); this is a top-down approach, where MDI represents the average departure of DNA methylation between initial tumor and recurrent tumor. Using this method, we found a significant MDI difference between the LMF-treated group and the TCGA control group (Fig. 2D).

We evaluated targets of LMF-induced DNA hypermethylation by gene ontology enrichment and REVIGO. We found that the most hypermethylated CpGs (Fig. 2B, orange dots) were associated with diverse pathways, including cell projection organization, neuronal differentiation, cell adhesion, and cell proliferation (Fig. 2E; Supplementary Figs. S3 and S4).

Finally, to further evaluate the clinical implications of using LMF + temozolomide, we studied changes in methylation of CpGs associated with MGMT after LMF treatment. A total of 163 CpGs were annotated to the MGMT locus out of a total of 207 contained within the EPIC array. In two of the six patient's tumors, we found increased DNA methylation in MGMT CpGs after treatment with LMF compared with their initial tumor (Supplementary Fig. S5).

## Discussion

Here we show that recurrent GBMs are epigenetically stable in comparison to the initial tumor with minimal loss of DNA methylation. Some DNA hypomethylation was appreciated but due to the small sample size this was not significant and this data is consistent with a larger study of 112 matched primary (*IDH* wild-type) GBM patients comparing initial tumor to recurrent tumor by Klughammer and colleagues (23); this group, using a different technique, reduced representation bisulfite sequencing, found additional loss of DNA methylation in some promoters of recurrent tumors, but no significant global hypomethylation in recurrent GBM. These results contrast what is seen in progression from *IDH*-mutant LGG to *IDH-*mutant GBM, where substantial DNA hypomethylation occurs in the *IDH*-mutant GBMs (9). We speculated the reason for this difference is a lower baseline global methylation in *IDH* wild-type GBM compared with *IDH-*mutant LGG. We show here that *IDH* wild-type GBM when compared with *IDH*-mutant LGG have significantly lower DNA methylation (Fig. 1C and D). Thus, we hypothesize that hypomethylated tumors, such as *IDH* wild-type GBMs can more easily remethylate during replication and thus less DNA hypomethylation occurs in late-replicating regions (12).

On the basis of the data that *IDH-*mutant gliomas have both increased global DNA methylation and survival compared with *IDH* wild-type gliomas, and that there is *in vivo* and *in vitro* data showing folate treatment can reduce growth of glioma cells, we tested whether the DNA methylome of *IDH* wild-type gliomas could be reprogrammed. We used LMF, for several reasons: first, folate or folic acid when taken by mouth is not efficiently transported into the brain; however, tetrahydrofolate forms such as LMF are (24). Moreover, LMF has shown clinical activity in two CNS clinical trials including a phase IV study of selective serotonin reuptake inhibitor–resistant depression (25, 26). Even though all patients in phase I had failed standard therapy with temozolomide, temozolomide was used because preclinical data showed folate treatment increased the sensitivity of temozolomide-induced apoptosis in glioma cells (13, 14).

We used recurrent *IDH* wild-type GBMs instead of recurrent *IDH*-mutant tumors in this phase I study for several reasons. Because *IDH* wild-type GBMs have worse prognosis than *IDH* mutants, our primary goal was to evaluate whether remethylation of the wild-type tumors could improve their survival and mimic the survival found in *IDH*-mutant GBMs. In addition, as we showed in Fig 1B and C, IDH wild-type GBMs are more hypomethylated at baseline than *IDH-*mutants, and thus to increase our ability to detect methylation change after remethylation, we used the more hypomethylated *IDH* wild-type gliomas. Finally, although the *IDH* mutation is the driver of hypermethylation, *IDH* mutants that retain this mutation still lose DNA methylation with progression from *IDH*-mutant LGG to *IDH*-mutant GBM, implying other defects in the methylation machinery or other factors in the methylation pathway go awry with progression to *IDH*-mutant GBM (9). We were concerned that such defects could interfere with LMF remethylation in *IDH* mutants, and thus, *IDH* mutants were excluded.

Our trial data shows that LMF was well tolerated and is safe. While we found no significant impact on survival in recurrent high-grade gliomas, we do show for the first time that the DNA methylome of *IDH* wild-type high-grade gliomas can be reprogrammed.

The limitation of this study is the sample size, as the phase I trial was not powered to detect a survival advantage. Likewise, the paucity of paired recurrent/autopsy GBM samples treated with LMF, limited our ability to identify the effects of tumor heterogeneity on LMFs ability to reprogram the DNA methylome (9, 11, 27–31).

In conclusion, we show here for the first time in a human study that the DNA methylome of high-grade glioma can be reprogrammed. Given the emerging role of epigenetic treatments in immuno-oncology, understanding the dynamics of epigenetic reprogramming will be essential and our work is a first step toward this goal (32).

## Supplementary Material

Figures S1-5, Table S1 Figure S1 shows progression-free survival of LMF-treated patients.
Figure S2 shows the genomic context of MDI after LMF treatment.
Figure S3 shows gene pathways impacted by LMF therapy.
Figure S4 shows gene ontology treemaps of pathways impacted by LMF treatment.
Figure S5 shows the impact of LMF treatment on MGMT methylation.
Table S1 shows the patient characteristics of autopsied patients.

## References

[1] Timp W , Bravo HC, McDonald OG, Goggins M, Umbricht C, Zeiger M, . Large hypomethylated blocks as a universal defining epigenetic alteration in human solid tumors. Genome Med 2014;6:61–71. 25191524 10.1186/s13073-014-0061-y PMC4154522

[2] Feinberg AP , Vogelstein B. Hypomethylation distinguishes genes of some human cancers from their normal counterparts. Nature 1983;301:89–92. 6185846 10.1038/301089a0

[3] Hansen KD , Timp W, Bravo HC, Sabunciyan S, Langmead B, McDonald OG, . Increased methylation variation in epigenetic domains across cancer types. Nat Genet 2011;43:768–75. 21706001 10.1038/ng.865 PMC3145050

[4] Berman BP , Weisenberger DJ, Aman JF, Hinoue T, Ramjan Z, Liu Y, . Regions of focal DNA hypermethylation and long-range hypomethylation in colorectal cancer coincide with nuclear lamina-associated domains. Nat Genet 2012;44:40–6. 10.1038/ng.969 PMC4309644 22120008

[5] Toyota M , Ahuja N, Ohe-Toyota M, Herman JG, Baylin SB, Issa JP, . CpG island methylator phenotype in colorectal cancer. Proc Natl Acad Sci USA 1999;96:8681–6. 10411935 10.1073/pnas.96.15.8681 PMC17576

[6] Noushmehr H , Weissenberger DJ, Diefes K, Phillips HS, Pujara K, Berman BP, . Identification of a CpG island methylator phenotype that defines a distinct subgroup of glioma. Cancer Cell 2010;17:510–22. 20399149 10.1016/j.ccr.2010.03.017 PMC2872684

[7] Brennan CW , Verhaak RG, McKenna A, Campos B, Noushmehr H, Salama SR, . The somatic genomic landscape of glioblastoma. Cell 2013;155:462–77. 24120142 10.1016/j.cell.2013.09.034 PMC3910500

[8] Turcan S , Rohle D, Goenka A, Walsh LA, Fang F, Yilmaz E, . IDH1 mutation is sufficient to establish the glioma hypermethylator phenotype. Nature 2012;483:479–83. 22343889 10.1038/nature10866 PMC3351699

[9] Mazor T , Pankov A, Johnson BE, Hong C, Hamilton EG, Bell RJ, . DNA methylation and somatic mutations converge on the cell cycle and define similar evolutionary histories in brain tumors. Cancer Cell 2015;28:307–17. 26373278 10.1016/j.ccell.2015.07.012 PMC4573399

[10] de Souza CF , Sabedot TS, Malta TM, Stetson L, Morozova O, Sokolov A, . A distinct DNA Methylation shift in a subset of Glioma CpG Island Methylator phenotypes during tumor recurrence. Cell Rep 2018;23:637–51. 29642018 10.1016/j.celrep.2018.03.107 PMC8859991

[11] Ceccarelli M , Barthel FP, Malta TM, Sabedot TS, Salama SR, Murray BA, . Molecular profiling reveals biologically discrete subsets and pathways of progression in diffuse glioma. Cell 2016;164:550–63. 26824661 10.1016/j.cell.2015.12.028 PMC4754110

[12] Zhou W , Dinh HQ, Ramjan Z, Weisenberger DJ, Nicolet CM, Shen H, . DNA methylation loss in late-replicating domains is linked to mitotic cell division. Nat Genet 2018;50:591–602. 29610480 10.1038/s41588-018-0073-4 PMC5893360

[13] Hervouet E , Debien E, Campion L, Charbord J, Menanteau J, Vallette FM, . Folate supplementation limits the aggressiveness of glioma via the remethylation of DNA repeats element and genes governing apoptosis and proliferation. Clin Cancer Res 2009;15:3519–29. 19451595 10.1158/1078-0432.CCR-08-2062

[14] Cartron PF , Hervouet E, Debien E, Olivier C, Pouliquen D, Menanteau J, . Folate supplementation limits the tumourigenesis in rodent models of gliomagenesis. Eur J Cancer 2012;48:2431–41. 22325970 10.1016/j.ejca.2012.01.002

[15] Quant EC , Wen PY. Response assessment in neuro-oncology. Curr Oncol Rep 2011;13:50–56. 21086192 10.1007/s11912-010-0143-y

[16] Aryee MJ , Jaffe AE, Corrada-Bravo H, Ladd-Acosta C, Feinberg AP, Hansen KD, . Minfi: A flexible and comprehensive bioconductor package for the analysis of Infinium DNA methylation microarrays. Bioinformatics 2014;30:1363–9. 24478339 10.1093/bioinformatics/btu049 PMC4016708

[17] Zhou W , Triche TJ, Laird PW, Shen H. SeSAMe: reducing artifactual detection of DNA methylation by Infinium BeadChips in genomic deletions. Nucleic Acids Res 2018;46:1–15. 30085201 10.1093/nar/gky691 PMC6237738

[18] Zhou W , Laird PW, Shen H. Comprehensive characterization annotation and innovative use of Infinium DNA methylation BeadChips probes. Nucleic Acids Res 2017;45:e22. 27924034 10.1093/nar/gkw967 PMC5389466

[19] Ritchie ME , Phipson B, Wu D, Hu Y, Law CW, Shi W, . limma powers differential expression analyses for RNA-sequencing and microarray studies. Nucleic Acids Res 2015;43:e47. 25605792 10.1093/nar/gkv007 PMC4402510

[20] Salas LA , Johnson KC, Koestler DC, O’Sullivan DE, Christensen BC. Integrative epigenetic and genetic pan-cancer somatic alteration portraits. Epigenetics 2017;12:561–74. 28426276 10.1080/15592294.2017.1319043 PMC5687331

[21] Phipson B , Maksimovic J, Oshlack A. missMethyl: an R package for analyzing data from Illumina's humanmethylation 450 platform. Bioinformatics 2016;32:286–8. 26424855 10.1093/bioinformatics/btv560

[22] Supek F , Bošnjak M, Škunca N, Šmuc T. REVIGO summarizes and visualizes long lists of gene ontology terms. PLoS One 2011;6:e21800. 21789182 10.1371/journal.pone.0021800 PMC3138752

[23] Klughammer J , Kiesel B, Roetzer T, Fortelny N, Nemc A, Nenning KH, . The DNA methylation landscape of glioblastoma disease progression shows extensive heterogeneity in time and space. Nat Med 2018;24:1611–24. 30150718 10.1038/s41591-018-0156-x PMC6181207

[24] Mattson RH , Gallagher BB, Reynolds EH, Glass D. Folate therapy in epilepsy. A controlled study. Arch Neurol 1973;29:78–81. 4577819 10.1001/archneur.1973.00490260022002

[25] Zajecka JM , Fava M, Shelton RC, Barrentine LW, Young P, Papakostas GI. Long-term efficacy, safety, and tolerability of L-methylfolate calcium 15 mg as adjunctive therapy with selective serotonin reuptake inhibitors: a 12-month, open-label study following a placebo-controlled acute study. J Clin Psychiatry 2016;77:654–60. 27035404 10.4088/JCP.15m10181

[26] Roffman JL , Petruzzi LJ, Tanner AS, Brown HE, Eryilmaz H, Ho NF, . Biochemical, physiological and clinical effects of l-methylfolate in schizophrenia: a randomized controlled trial. Mol Psychiatry 2018;23:316–22. 28289280 10.1038/mp.2017.41 PMC5599314

[27] Wang Q , Hu B, Hu X, Kim H, Squatrito M, Scarpace L, . Tumor evolution of glioma-intrinsic gene expression subtypes associates with immunological changes in the microenvironment. Cancer Cell 2017;32:42–56. 28697342 10.1016/j.ccell.2017.06.003 PMC5599156

[28] Kim J , Lee IH, Cho HJ, Park CK, Jung YS, Kim Y, . Spatiotemporal evolution of the primary glioblastoma genome. Cancer Cell 2015;28:318–28. 26373279 10.1016/j.ccell.2015.07.013

[29] Consortium G . Glioma through the looking GLASS: molecular evolution of diffuse gliomas and the Glioma Longitudinal Analysis Consortium. Neuro Oncol 2018;20:873–84. 29432615 10.1093/neuonc/noy020 PMC6280138

[30] deCarvalho AC , Kim H, Poisson LM, Winn ME, Mueller C, Cherba D, . Discordant inheritance of chromosomal and extrachromosomal DNA elements contributes to dynamic disease evolution in glioblastoma. Nat Genet 2018;50:708–17. 29686388 10.1038/s41588-018-0105-0 PMC5934307

[31] Chen X , Wen Q, Stucky A, Zeng Y, Gao S, Loudon WG, . Relapse pathway of glioblastoma revealed by single-cell molecular analysis. Carcinogenesis 2018;39:931–36. 29718126 10.1093/carcin/bgy052 PMC6248540

[32] Topper MJ , Vaz M, Marrone KA, Brahmer JR, Baylin SB. The emerging role of epigenetic therapeutics in immuno-oncology. Nat Rev Clin Oncology 2020;17:75–90. 10.1038/s41571-019-0266-5 PMC7254932 31548600

