# Supplementary material for: Phase I Study of High-Dose l-Methylfolate in Combination with Temozolomide and Bevacizumab in Recurrent IDH Wild-Type High-Grade Glioma
Source: Cancer Res Commun. 2022 Jan 5;2(1):1–9. doi: 10.1158/2767-9764.CRC-21-0088 (PMC8983000; doi:10.1158/2767-9764.CRC-21-0088)

**Supplemental Material: Phase I Study of High-Dose L-methylfolate in Combination with Temozolomide and Bevacizumab in Recurrent IDH wild type High-Grade Glioma**

Lucas A. Salas^1^, Thomas G. Stewart^2^, Bret C. Mobley^3^, Chengwei Peng^4^, Sudan N. Loganathan^5^, Yanjun Ma^6^, Jialiang Wang^5^, Mitchell S. Berger^7^, Devin Absher^8^, Yang Hu^9^, Paul L. Moots^10^, Brock C. Christensen^1,11^§, Stephen W. Clark^10,12^§*

^1^Department of Epidemiology, Geisel School of Medicine at Dartmouth, Hanover, NH

^2^Department of Biostatistics, Vanderbilt University Medical Center, Nashville, TN

^3^Department of Pathology, Vanderbilt University Medical Center, Nashville, TN

^4^Department of Medicine, Yale Medical School, New Haven, CT

^5^Department of Neurosurgery, Vanderbilt University Medical Center, Nashville, TN

^6^Tennessee Oncology PLLC, Nashville, TN

^7^Department of Neurosurgery, UCSF, San Francisco, CA

^8^HudsonAlpha, Huntsville, AL

^9^CD Genomics, Shirley, NY

^10^Division of Neuro-Oncology, Vanderbilt University Medical Center, Nashville, TN

^11^Department of Molecular and Systems Biology, Geisel School of Medicine at Dartmouth, Hanover, NH

^12^Sir Gallahad Labs, Nashville, TN

§These authors jointly supervised this work

*corresponding author

# **Supplemental Figure 1. Progression-free survival of LMF-treated patients.**


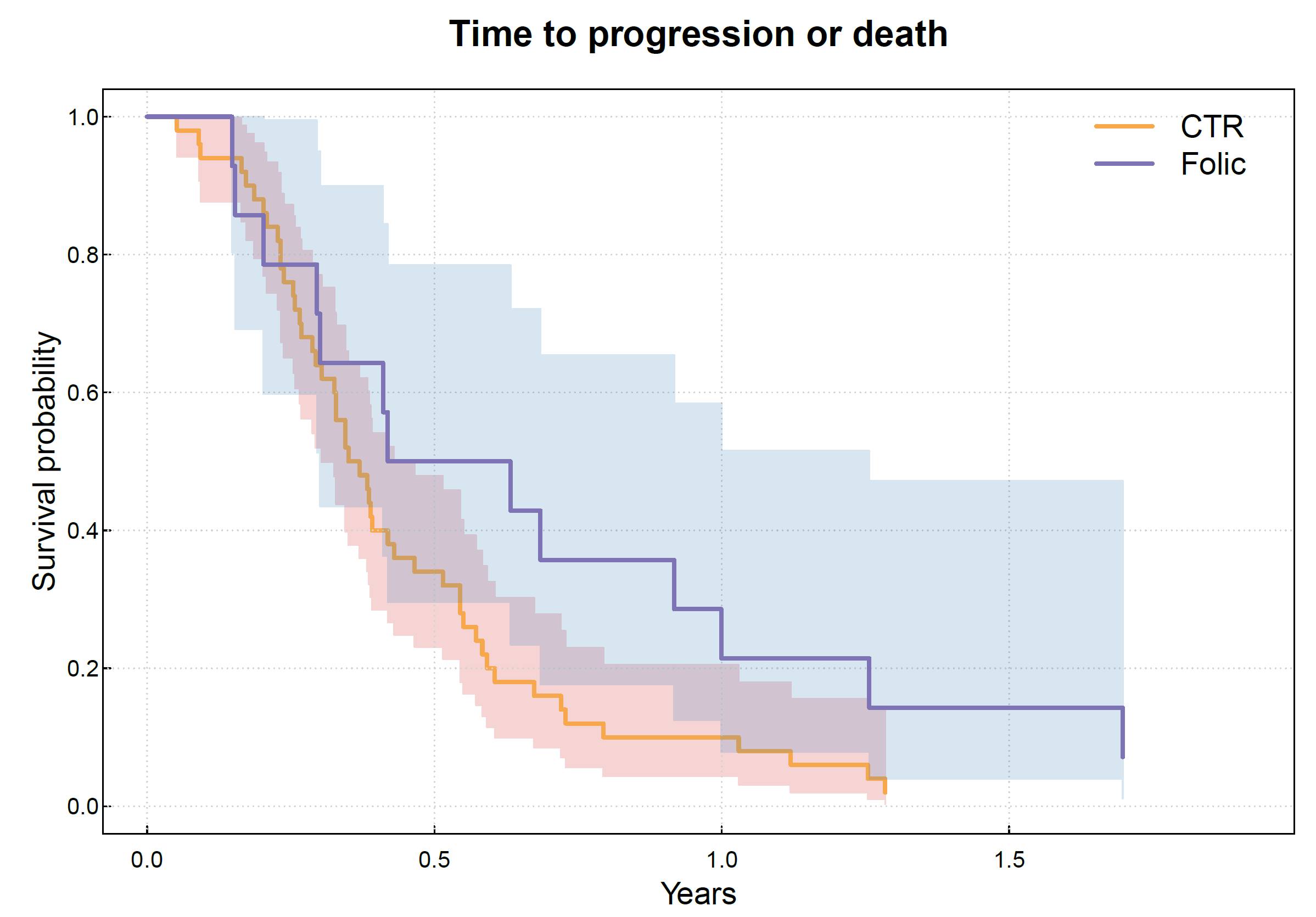


LMF-treated patients (blue line) versus historical control group treated with bevacizumab alone (red line). The 95% CI is represented by the blue and red shaded areas. See Supplementary Table 5 for patient characteristics, including n value.

# **Supplemental Figure 2. The genomic context of the methylation dysregulation index after treatment with LMF vs controls.**


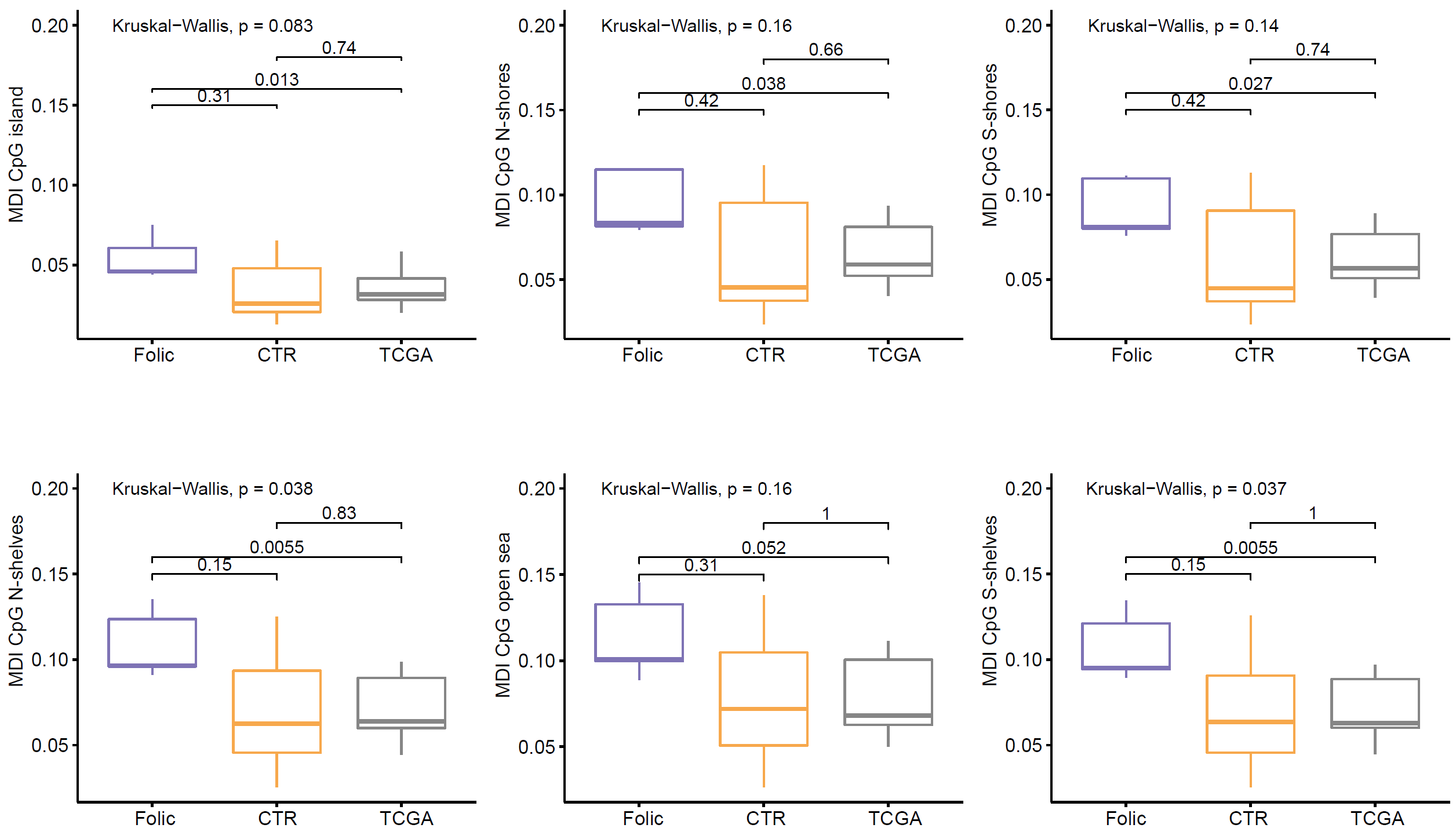


Comparison of the DNA methylation dysregulation index (MDI) between LMF-treated patients (Folic), TCGA paired samples (TCGA), and our institutional paired controls (CTR). Box plot elements as in Figure 2C.

# **Supplemental Figure 3. Pathways impacted by LMF therapy.**


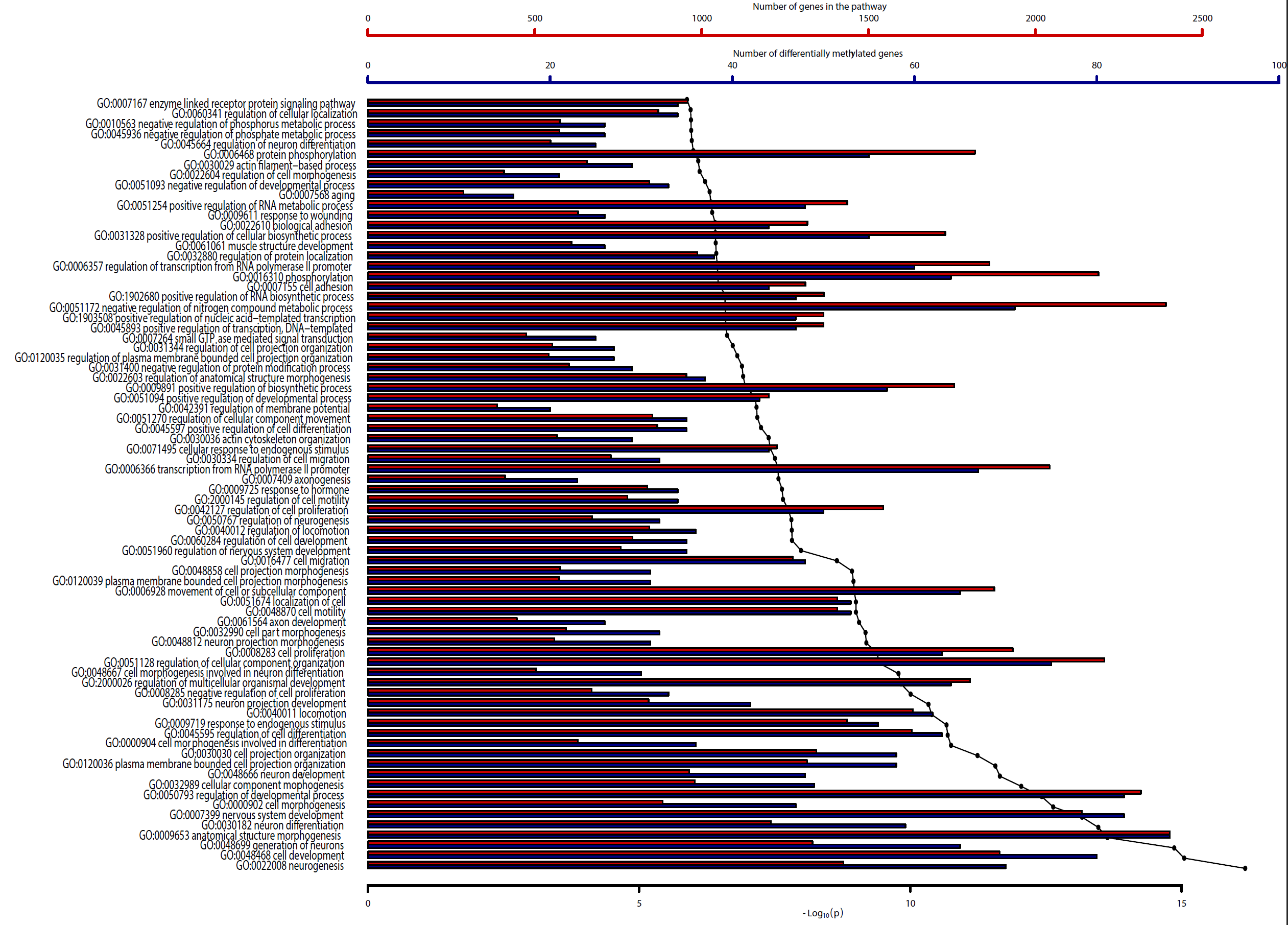


Gene ontology (GO) analysis of pathways impacted by the 645 hypermethylated (>0.2 Δ𝛃) CpGs in LMF treated patients. Barplot of the top results from GO analysis of pathways, which have genes significantly hypermethylated after therapy with LMF in recurrent IDH†-wt GBMs (all P-values <1.0E-07).

# **Supplemental Figure 4. REVIGO Gene ontology treemap of pathways impacted by LMF treatment.**


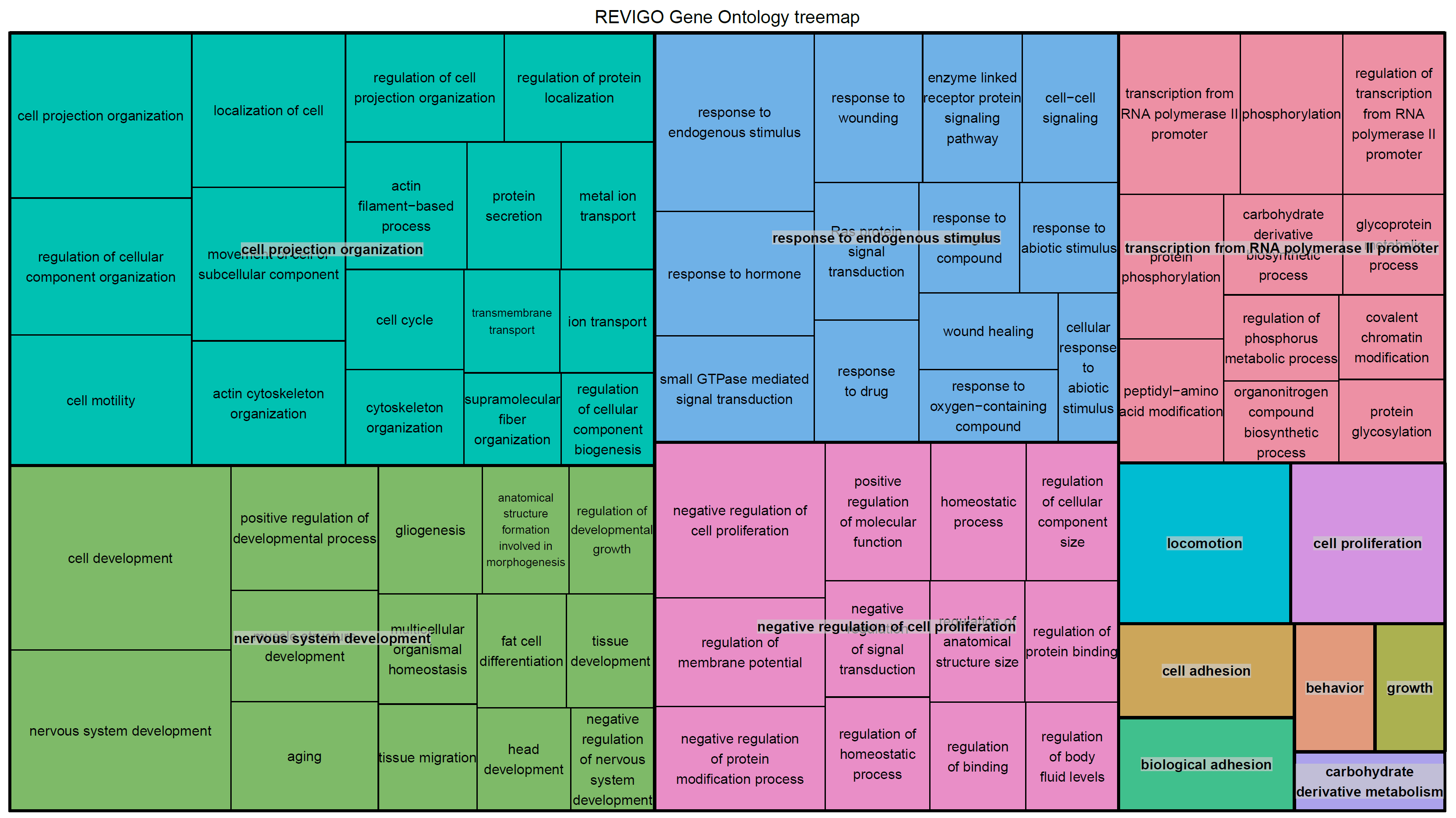


REVIGO treemap summarizing gene ontology biological process categories over-represented in LMF treated tumors. Over-represented categories with *P* values less than 0.001 (74- terms) were used to generate a treemap colored by functional category. The size of each rectangle is proportional to the p value for that category.

# **Supplemental Figure 5. Impact of LMF on MGMT methylation.**


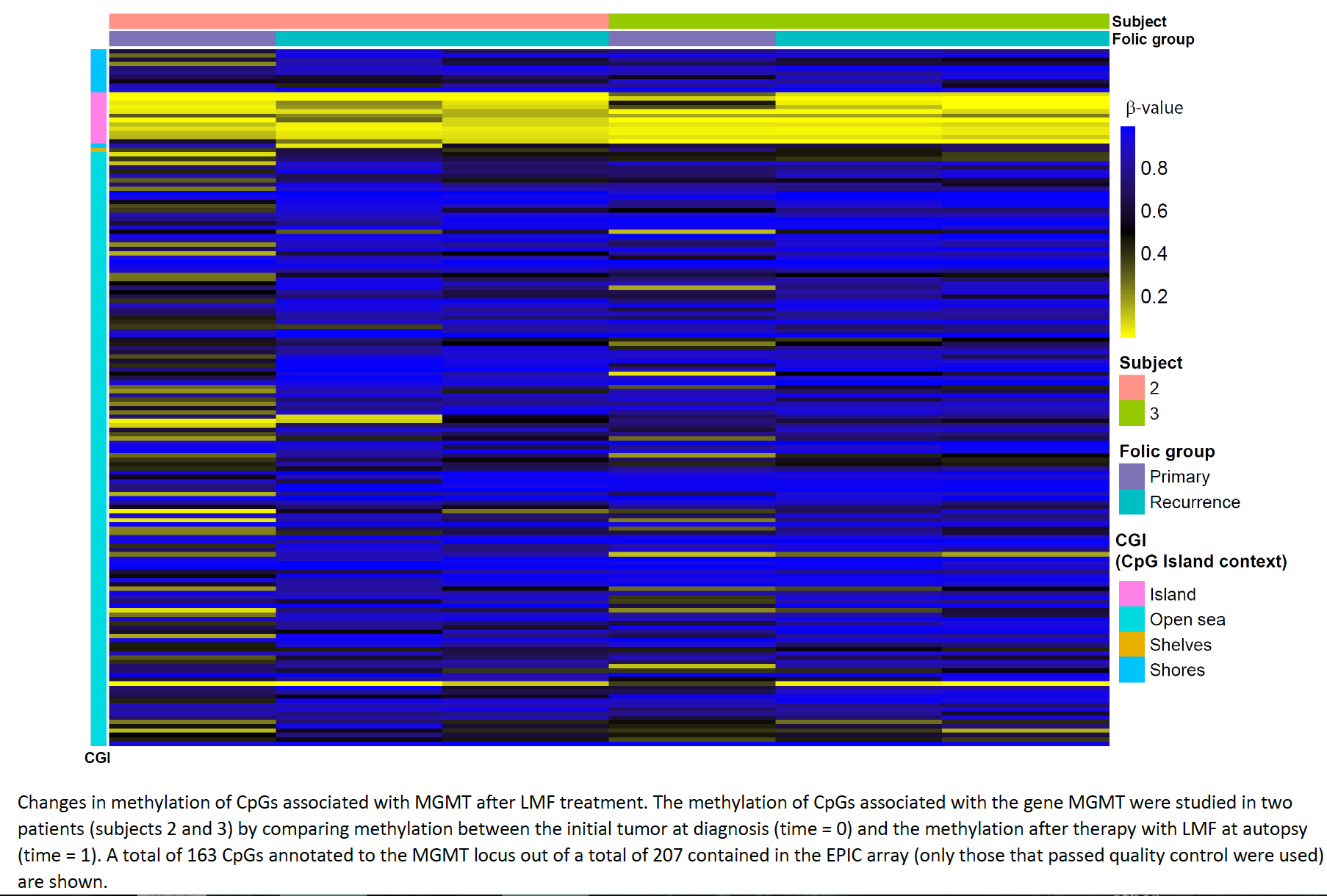


# **Supplemental Table 1. Patient characteristics of autopsied patients.**


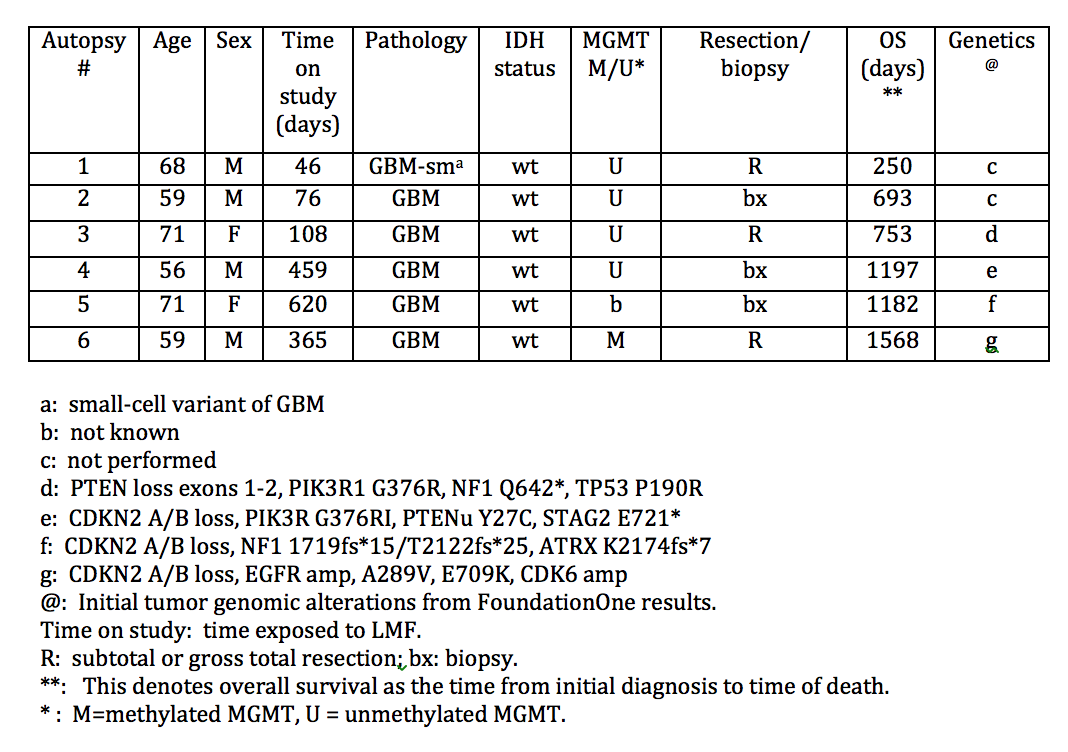

Supplement: Figures S1-5, Table S1 — Figure S1 shows progression-free survival of LMF-treated patients. Figure S2 shows the genomic context of MDI after LMF treatment. Figure S3 shows gene pathways impacted by LMF therapy. Figure S4 shows gene ontology treemaps of pathways impacted by LMF treatment. Figure S5 shows the impact of LMF treatment on MGMT methylation. Table S1 shows the patient characteristics of autopsied patients. [file crc-21-0088-s01.docx]
